# Bipolar Disorder Affects Behavior and Social Skills on the Internet

**DOI:** 10.1371/journal.pone.0079673

**Published:** 2013-11-11

**Authors:** Thaís Martini, Letícia Sanguinetti Czepielewski, Adam Fijtman, Leonardo Sodré, Bianca Wollenhaupt-Aguiar, Caroline Silveira Pereira, Mireia Vianna-Sulzbach, Pedro D. Goi, Adriane Ribeiro Rosa, Flavio Kapczinski, Maurício Kunz, Marcia Kauer-Sant'Anna

**Affiliations:** 1 Programa de Pós-Graduação em Ciências Médicas: Psiquiatria, Universidade Federal do Rio Grande do Sul, Porto Alegre, Brazil; 2 Laboratory of Molecular Psychiatry, Hospital de Clínicas de Porto Alegre, Universidade Federal do Rio Grande do Sul, Instituto Nacional de Ciências e Tecnologia Translacional em Medicina, Porto Alegre, Brazil; 3 Programa de Pós-Graduação em Ciências Biológicas: Bioquímica, Federal University of Rio Grande do Sul, Porto Alegre, Brazil; 4 Hospital de Clínicas de Porto Alegre, Faculdade de Medicina, Departamento de Psiquiatria e Medicina Legal - Universidade Federal do Rio Grande do Sul, Porto Alegre, Brazil; University of São Paulo, Brazil

## Abstract

**Background:**

Bipolar disorder (BD) is a significant cause of functional, cognitive, and social impairment. However, classic studies of functioning and social skills have not investigated how BD may impact behavior on the Internet. Given that the digital age has been changing the way people communicate, this study aims to investigate the pattern of Internet use in patients with BD.

**Methods:**

This cross-sectional study assessed 30 patients with BD I or II and 30 matched controls. Patients were not in an acute mood episode, according to DSM-IV. A standard protocol examined sociodemographic variables and social behavior on the Internet, assessed by Facebook number of friends (FBN) and lifetime estimated number of offline contacts (social network number, SNN).

**Results:**

SNN (p<0.001) and FBN (p = 0.036) of patients with BD were significantly lower than those of controls. Also, variables related with Internet use were significantly lower in patients, e.g., close contacts on Facebook (p = 0.021), Internet experience (p = 0.020), and knowledge of terms associated with social networking sites (p = 0.042). Also, patients showed lower rates of the expected pattern of Internet use (based on their age generation), including a poorer knowledge of SNS (p = 0.018) and a lower frequency of Internet use (p = 0.010).

**Discussion:**

This study suggests that patients with BD show smaller social networks both in real-world settings and on the Internet. Also, patients tend to use the Internet and social networking sites less frequently and show a poorer knowledge of Internet and social media than healthy controls, below the expected for their generation. These significant differences between patients and controls suggest that the effects of BD on social relationships and functioning extend to electronic media.

## Introduction

Bipolar disorder (BD) is associated with significant functional [1]–[3], cognitive [4], and social [5]–[7] impairment. As the disease progresses, patients often show significant deficits in social skills and functioning, which have been shown to be associated with cognitive impairment [4], [8]. Indeed, poor functioning is associated with both social and family burden [4], and may impact the social support network [5]. Social relationships play an important role in maintaining well-being, as they determine social competence to keep a professional life and healthy relationships [9]–[11]. Also, people with adequate social networks have a 50% greater likelihood of survival [12]. Social disengagement is associated with poor quality of life and physical/psychological health [13]; diversified social networks, in turn, have been associated with improved quality of life [14], [15].

The digital age has imposed significant changes on social relationships [16]. Over 2.3 billion (i.e., 1 in 3) people in the world use the Internet to consume information and to communicate [17]. In particular, the use of social networking sites (SNS) has been increasing at fast rates [18], [19]. At present, Facebook is the biggest SNS in the world, with more than a billion monthly active users [20]. As well, in Brazil, Facebook network site is the most popular according to a national survey in 2012 (IBOPE) [21], [22]. SNS have changed the way people communicate, consume/share information, and receive social support [23]. Social groups have changed, and new dynamics of interaction emerge [24]. The so-called digital natives, people born during or after the advent of the Internet, have many opportunities to fit into different kinds of digital social interaction and show great familiarity with technological devices. Each specific generation has been shown to present different patterns of behavior, according to their age, environment, and culture [25]–[27], e.g., the Baby Boomers (people born between 1943 and 1960), the X generation (1961 and 1981), and the Y generation, also known as the Millennials (1982 and 2001).

Classic studies have shown a strong association between mood/health and social network. For example, the social contagion theory has suggested a significantly impact of social networks on smoking [28], obesity [29], depression [30], and happiness [31]. On the Internet, even though people's connections on SNS seem to cause behavioral changes [26], few articles have investigated the relationships between Internet-based social networks and psychiatric symptoms. Some reports have suggested that Internet and television may be associated with social isolation [13], depression [32], [33] (with controversial reports [34]), narcissism [35], addiction [36]–[38], and changes in child development [39].

Despite extensive research into SNS in general, its use among patients with BD has not been investigated. A literature review has revealed only studies describing the prevalence of psychiatric symptoms in subjects with problematic Internet use [40], [41] and one case report suggesting potential benefits of Facebook in the rehabilitation of a patient with BD [42]. It is likely that the functional impairment associated with BD may also impact behavior also on the Internet [9]. However, to our knowledge, this is the first study to compare patterns of Internet use in patients with BD and controls.

## Methods

### Subjects

A total of 30 patients with BD I and II according to criteria from the Diagnostic and Statistical Manual of Mental Disorders, 4th edition (DSM-IV) were recruited among subjects treated at the Bipolar Disorder Program at Hospital de Clínicas de Porto Alegre, Federal University of Rio Grande do Sul, in Porto Alegre, southern Brazil. All patients were interviewed by a trained psychiatrist (using SCID) and were not in an acute mood episode according to DSM-IV criteria. Exclusion criteria were presence of neurological disease or any physical condition that could restrict the patient's ability to use a computer. We also included 30 controls, matched by gender, age, and years of education. Controls were screened using the SCID (non-patient version); those selected for inclusion also completed the FAST and the standard protocol described below. Controls did not report lifetime psychiatric or neurodegenerative disorders and were not on psychiatric medication. They did not fulfill criteria for any psychiatry diagnostic and they were not in an acute episode.

### Ethics

The study protocol was approved by the Research Ethics Committee of Hospital de Clínicas de Porto Alegre and Federal University of Rio Grande do Sul, and all participants signed an informed consent form before entering the study. This clinical investigation had been conducted according to the principles expressed in the Declaration of Helsinki. None of the scales or questionnaires used in the study abashed or intimidated participants, and recruiters were instructed to monitor any discomfort with the questionnaire.

### Functioning, quality of life, intellectual ability, and clinical interview

Patient evaluation consisted of a psychiatric interview including diagnostic assessment (Structured Clinical Interview for DSM-IV, SCID) and symptom rating using the HAM-D [43] and YMRS [44] scales. Functioning was assessed using the Functional Assessment Staging Tool (FAST) [45], and general intellectual ability was assessed from the weighted scores of the subtests of vocabulary and block design from the WAIS-III [46], [47]. The Satisfaction With Life Scale (SWLS) [48] was also used to measure quality of life in both groups.

### Sociodemographic data, Internet use, and SNS

A standard protocol examined sociodemographic variables (age, years of education, gender, economic class, marital status, and occupation), as well as variables related to Internet use patterns. Economic class was categorized using a scale validated in Brazil [49]. The interval between psychiatric assessment and protocol interview was a maximum of three days.

For the assessment of Internet use patterns, subjects were evaluated using a standard protocol divided into five categories: Social Network, SNS, Facebook, Internet, and Technology. The following variables were adapted from the Social Network Index (SNI) scale [15], according to Bickart et al. [50] and Kanai et al. [51]: social network groups (SNG), which estimates to how many different social groups (work, family, school) a person is connected; the social network number (SNN), which estimates the total number of social contacts offline; and social network – two weeks (SNTW), which estimates the number of offline acquaintances that a person contacts over two weeks.

In addition, some variables of the questionnaire were adapted from the Pew Internet and American Life Project [16], [52], [53] and are described in detail in Protocol S1. Briefly, lifetime SNS use. e.g., LinkedIn, Facebook, Orkut, and Twitter (number of SNS); frequency of use of SNS (frequency of SNS). To check a possible bias regarding the use of other social networks, the questionnaire included other social networks: Orkut, Twitter and LinkedIn. However, the Facebook was the most used social network site, in line with a recent report of IBOPE (2013) and Socialbacker (2013). These instruments were translated into Portuguese and adapted by trained researchers in a pilot study.

The number of contacts added to Facebook, or Facebook number of friends (FBN), has also been used in previous studies [49], [50], [54] and was assessed in our sample. Also, the questionnaire covered the following items: experience on Facebook (FB), close contacts on FB, acquaintances on FB, virtual friendships on FB, frequency of FB use, Internet experience, Internet use, frequency of Internet use (daily), Internet experience on devices, and number of digital devices.

Finally, a list of 12 terms commonly used in SNS was assembled and used to assess the subjects' familiarity with SNS (they were asked to inform how many terms of the list they had already heard or used – SNS terms). In a second stage, the definitions of each term were read, and subjects were asked to cross-check them with a list of terms. The number of correct answers on this test was used as a score (correct SNS).

We included two variables to assess whether the subjects showed the expected behavior on the Internet according to their age generation (Millennials or Y, X, and Baby Boomer) [16], [19], [22], [23], namely, knowledge of SNS (G-ESNS) and frequency of Internet use (G-EFI). According to the data reviewed, we determined that Millennials, also called Gen Y (8 terms), are expected to know more SNS terms than Gen X (5 terms) and Baby Boomers (1 term). Then, we defined an expected “Correct SNS” score range for each generation. Based on these ranges, the variable G-ESNS was a dichotomous variable (performance was within range expected for generation or not). In line with the approach adopted for G-ESNS, the expected frequency of Internet use according to each subject's generation (G-EFI) was measured using scores previously determined. Millennials were expected to use the Internet daily, Gen X at least three times a week, and Baby Boomers one time in two weeks on average (see Protocol S1).

### Statistics

First, the Kolmogorov-Smirnov test was used to determine normality and validity of all variables. Subsequently, differences between patients and controls were assessed using the chi-squared test for categorical variables and the independent t-test and Mann-Whitney U-test for continuous variables, as appropriate. Exploratory correlations were examined using Pearson and Spearman correlation coefficients, according to normality status. All non-parametric data were expressed as median (interquartile range), and parametric data as mean (standard deviation). Analyses were performed using the Statistical Package for the Social Sciences (SPSS) version 19.0 for Windows (SPSS Inc., Chicago, Illinois, USA). Significance was set at p<0.05.

## Results

### Sociodemographic and clinical variables

Age of BD patients ranged between 18 and 55 years, with a mean of 35.33±10.71 years; 23 (76.70%) were female. Matched controls presented similar age (18 to 55 years, mean: 34.80±10.70 years; p = 0.849) and the same number of female patients (23, 76.70%; p = 1.000). The groups were also statistically similar in terms of years of education (p = 0.736) and economic class (p = 0.409), but marital status was different (p = 0.038) (Table 1).

**Table 1:** Demographic, functioning, quality of life, intellectual ability and clinical characteristics.

Category	Subcategory	Subcategory	Patients (n = 30)	Controls (n = 30)	÷^2^ a, t^b^, or U^c^	p
**Demographic**	Age		35.33 (10.71)	34.80 (10.70)	0.19^b^	0.849
	Gender	Female	23 (76.70)	23 (76.70)	0.00^a^	1.000
	Years of education		12 (5)	11 (4)	427.50^c^	0.736
	Marital status	Married	12 (40.00)	20 (66.70)	4.286^a^	*0.038* *
	Economic class				1.78^a^	0.409
		A	2 (6.70)	3 (10.00)		
		B	14 (46.70)	18 (60.00)		
		C	14 (46.70)	9 (30.0)		
	Generations					
		Millennials	11 (36.70)	11 (36.70)		
		Generation X	17 (56.70)	17 (56.70)		
		Baby Boomers	2 (6.70)	2 (6.70)		
**Functioning**	FAST		22.67 (13.01)	8.77 (6.80)	5.18^b^	*<0.01* *
		Autonomy	2.86 (2.17)	0.93 (1.33)	4.14^b^	*<0.01* *
		Occupational functioning	4.06 (2.99)	1.16 (1.51)	4.73^b^	*<0.01* *
		Cognitive functioning	6.20 (3.35)	2.96 (1.93)	4.56^b^	*<0.01* *
		Financial issues	2.30 (2.21)	0.93 (1.17)	2.98^b^	*0.016* *
		Interpersonal relationships	4.60 (3.42)	1.50 (2.01)	4.27^b^	*<0.01* *
		Leisure time	2.40 (2.07)	1.23 (1.30)	2.60^b^	*0,031* *
**Quality of life**	SWLS		20.00 (14.00)	28.00 (8.00)	145.00^c^	*<0.01* *
**Intellectual ability**			95.00 (15.00)			
**Clinical**						
	YMRS		1.00 (3.00)			
	HAM-D		5.00 (4.00)			
	Duration of illness		17.73 (10.08)			
	Total number of episodes		11.00 (16.00)			
	Number of hospitalizations		2.00 (3.00)			
	Number of suicide attempts		2.00 (3.00)			
	Diagnosis					
		Bipolar I	26 (86.70)			
		Bipolar II	4 (13.33)			
	Episode onset					
		Mania	5 (16.70)			
		Hypomania	5 (16.70)			
		Depression	20 (66.70)			

a Chi-square test; ^b^ independent t-test; ^c^ Mann-Whitney test.

* p<0.05.

YMRS: Young Mania Rating Scale; HAM-D: Hamilton Depression Rating Scale; FAST: Functional Assessment Screening Tool; SWLS: Satisfaction With Life Scale.

Analysis of clinical characteristics revealed that most patients were bipolar I (N = 26, 86.70%), showed depressive as episode onset (N = 20, 66.70%), and had the illness for 17.73±10.08 years. Median scores on HAM-D were 5.00, and on YMRS, 1.00. Also, all patients showed a YMRS less than 7 and a HAM-D less than 12 (mean scores are described in Table 1). Results for sociodemographics and clinical variables are described in Table 1.

### Functioning, quality of life and intellectual ability

Functioning scores (FAST) were lower in patients (Table 1): they reported having greater difficulty performing routine tasks when compared to controls (U = 152.00, p<0.001). Patients also showed lower levels of satisfaction (SWLS) with their lives (U = 145.00, p<0.001). Also, patients presented an intellectual ability mean of 95.00 (15.00), controls were not assessed. FAST subitems (Table 1) were also lower in patients: autonomy (t = 4.14, p<0.001); occupational functioning (t = 4.73; p<0.001); cognitive functioning (U = 173.00, p<0.001); financial issues (U = 294.00, p = 0.016); interpersonal relationships (U = 181.50, p<0.001) and leisure time (U = 308.00, p = 0.031).

### Internet and SNS

**Figure 1:**
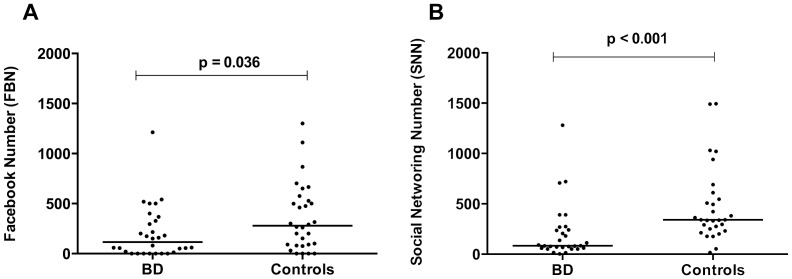
Social Networking Online and Offline in BD patients. **A**. The Facebook number of friends (FBN) in patients and controls. **B**. The social networking number or lifetime total number of friends (SNN) in patients and controls.

The variables describing patterns of Internet use (Table 2) showed significant differences between patients and controls. Patients had significantly lower SNN and FBN results than controls (U = 177.50, p<0.001, and U = 308.50, p = 0.036, respectively), shown in Figure 1.

**Table 2:** Internet and social network site characteristics.

Category	Subcategory	Patients (n = 30)	Controls (n = 30)	÷^2^ a, t^b^, or U^c^	p
**Social network**	SNG	5.00 (2.00)	4.00 (4.00)	332.50^c^	0.163
	SNTW	29.00 (43.00)	38.00 (50.00)	321.50^c^	0.124
	SNN	84.00 (195.00)	340.00 (338.00)	177.50^c^	*<0.01* *
**Social network sites (SNS)**	G-ESNS	13 (43.33)	22 (73.33)	5.55^a^	*0.018* *
	SNS terms	8.00 (7.00)	10.00 (4.00)	313.50^c^	*0.042* *
	Correct SNS	5.50 (6.00)	7.00 (5.00)	334.00^c^	0.084
	Number of SNS	2.00 (2.00)	2.00 (2.00)	307.50^c^	0.731
	Frequency of use of SNS (daily)	13 (43.33)	20 (66.70)	3.30^a^	0.069
**Facebook**	FBN	115.00 (328.00)	279.00 (452.00)	308.50^c^	*0.036* *
	Experience on Facebook	4.00 (5.00)	5.00 (3.00)	375.50^c^	0.263
	Close contacts on Facebook	15.00 (25.00)	24.50 (16.00)	295.00^c^	*0.021* *
	Acquaintances on Facebook	0.00 (5.00)	5.00 (5.00)	304.50^c^	*0.020* *
	Virtual friendships	3.00 (5.00)	4.00 (5.00)	427.00^c^	0.709
	Frequency of use of Facebook	8.00 (15.00)	14.00 (11.00)	341.50^c^	0.107
**Internet**	Internet use (yes)	26 (86.70)	27 (90.00)	0.16^a^	0.688
	Frequency of Internet use (daily)	20 (66.70)	26 (86.70)	3.35^a^	0.067
	Experience on Internet	10.47 (6.80)	14.73 (6.95)	−2.40^b^	*0.020* *
	G-EFI	17.00 (56.70)	26 (86.70)	6.64^a^	*0.010* *
**Technology**	Internet experience on devices	1.00 (1.00)	2.00 (0.00)	320.00^c^	*0.036* *
	Digital devices	3.00 (1.00)	3.00 (2.00)	290.00^c^	*0.014* *

a Chi-squared test; ^b^ independent t-test; ^c^ U Mann-Whitney test.

* p<0.05;

SNG: lifetime number of social network groups; SNTW: number of social network contacts contacted at least once over two weeks; SNN: social network number or lifetime total number of friends; G-ESNS: expected knowledge of SNS terms according to generation; SNS terms: familiarity with social network site terms; Correct SNS: number of correct answers in the social network site term test; Number of SNS: lifetime number of social network sites used; Frequency of use of SNS: frequency of use of social network sites; FBN: Facebook number of friends; Close contacts on Facebook: close contacts added to Facebook; Acquaintances on Facebook: people seen once added to Facebook; Virtual friendships: people never seen offline but added to Facebook; Frequency of use of Facebook: frequency of activities on Facebook;Frequency of Internet use: frequency of use of the Internet; G-EFI: expected frequency of Internet use according to generation; Digital devices: number of technological devices.

On Facebook, patients had fewer close contacts (U = 295.00, p = 0.021) and fewer acquaintances (U = 304.50, p = 0.020) when compared with controls. No correlation was found among patients between FBN and HAM-D (r = −0.017, p = 0.929) or YMRS (r = 0.334, p = 0.77).

Patients showed lower scores of familiarity with SNS terms than controls (U = 313.50, p = 0.042). They were also less experienced in the use of the Internet (t = −2.40, p = 0.020) and in the use of Internet devices (U = 320.00, p = 0.036). Patients also had fewer digital devices than controls (U = 290.00, p = 0.014).

Expected knowledge of SNS terms according to generation was significantly lower in patients than in controls (χ^2^ = 5.55; p = 0.018). In the same way, the expected frequency of Internet use according to generation was also lower in patients than controls (χ^2^ = 6.64; p = 0.010).

In addition we checked a correlation between the main outcome (FBN), with HAM-D and YMRS scores and it was not significant (FBN x HAM-D, r  = −0,017, p = 0,92; FBN x YMRS, r  =  0.33, p = 0.07). Also, while in controls there was no correlation between subitens of FAST (autonomy, occupational functioning, cognitive functioning, financial issues, interpersonal relationships, leisure time) and FBN and SNN (p>0.05), in patients with BD, cognition domain of FAST was significantly connected with SNN (p = 0.02) and also leisure time presented a correlation with SNN (p = 0.032).

## Discussion

To our knowledge, this is the first study to examine Internet and SNS use in BD patients compared with healthy controls. In order to enable an approach that could bridge the gap between psychiatry and Internet culture, a new protocol was developed from scratch (see Protocol S1). Our results suggest that patients with BD have poorer social networks both offline (SNN) and via Internet (FBN) than controls. It is conceivable that the cognitive impairment associated with BD be a mediator at least in part of these findings, however future studies should include a cognitive battery to confirm this. Nevertheless, previous studies reported that frequent Internet use is associated with cognitive abilities, mainly with executive function [55], [56]. Moreover, a recent work found a positive correlation between computer use and executive function that was seen even after controlling for basic intellectual ability [57]. People with high need for cognition are more experienced on Internet and use it longer [58]. Based on exploratory analysis of FAST subitems it seems that cognitive domain was associated with social network offline, but not online; which raises an interesting difference between online and offline social behavior and deserve further investigation in future studies.

Studies have linked amygdala volume and offline social network size, and brain regions responsible for social perception have been studied in relation to online social network size [50], [51], [59]. BD patients have enlarged amygdala [60], and reduced prefrontal structures [61]. The fact that orbital prefrontal cortex volume correlates with social cognitive competence [62] may help understand, from neurophysiological point of view, the significance of the data found in this study.

Previous studies have shown a significant relationship between the size of some brain structures and the number of people with whom an individual interacts in a group [63]. The social brain hypothesis states that the higher the number of members in a group, the higher the amount of information to be processed, with an influence on brain size. According to the hypothesis, this relationship imposes a limit that prevents people from expanding the network: 150 contacts at a time. The following question has guided some studies [14]: do better cognitive skills and higher intelligence regulate our social cognition (related to brain size), or does social cognition enable or influence our cognitive ability and intelligence?

Even though subsyndromal symptoms may contribute toward a reduced use of the Internet, no correlation was observed between FBN and HAM-D scores, suggesting that BD affects FBN regardless of the presence of depression. Economic class was not different between the two groups, so our results suggest that patients with BD were not interested in using the Internet and SNS. In addition, most patients had a computer and Internet access at home.

The significant differences between patients and controls found in our study suggest that the diagnosis of BD may affect social relationships and functioning also on electronic media. In this scenario, cognitive deficit emerges as a possible explanation, reflecting a poorer understanding of the Internet and its use. On the one hand, excessive use of the Internet and SNS is clearly questionable; on the other hand, not using it can also be harmful, because of the huge social loss there involved. In addition, not using SNS affects these patients' social support network, which has been proving to be an important treatment adjuvant [4], [7], [9]. Noteworthy is the fact that controversy remains as to whether heavy Internet use (perhaps approaching addiction) is or not associated with decreased social support. In our BD patients, no correlation was observed between internet use and social network.

Some of our findings replicate those of previous studies showing a poorer functioning in patients (FAST) [45] and lower levels of satisfaction with life (SWLS) [64]. At the sociodemographic level, controls were more frequently married, which reinforces the poor ability of BD patients to maintain an active social network. Taken together, these results highlight that there is social and functional impairment in BD, even when patients are not in an acute episode, which might be targeted by early intervention strategies.

These results should be interpreted in the light of some limitations. Even though subsyndromal symptoms may contribute toward a reduced use of the Internet, no correlation was observed between FBN and HAM-D scores, suggesting that BD affects FBN regardless of the presence of subsyndromal depression. However, we could not completely exclude the effect of subsyndromal symptoms on interest on internet when comparing to controls. Also, we did not include scales to assess cognition and general intellectual ability in both groups, which could possibly reveal a connection between the cognitive impairment and their knowledge of the Internet. The assumption that the deficit would be mediated by the cognitive impairment associated with BD progression deserves further investigation. Also, the analysis of such data could perhaps shed some light on identifying the exact stage of the disease at which the patient becomes socially impaired and whether such impairment overlaps with decreases in their cognitive or functional abilities.

In sum, our data potentially add a new (digital) domain to the classic studies of functioning and social skills in BD. More importantly, though, there is a worrying trend: patients with BD have fewer friends both in real-world settings and on the Internet, and their online behavior (in terms of frequency and knowledge of terms used in SNS) does not match that of healthy individuals in the same age group. The lack of interest of these patients in the Internet and in SNS further impairs their socialization and communication, wasting a valuable opportunity to improve their learning skills and their access to information. Further research should clarify which brain areas and cognitive skills are involved in Internet behavior in BD and help ensure healthy social opportunities for patients.

## Supporting Information

Protocol S1 **Interview on Internet and social network sites (SNS).** A detailed description of the variables assessed.
(DOC)
