# Supplementary material for: Bipolar Disorder Affects Behavior and Social Skills on the Internet
Source: PLoS One. 2013 Nov 11;8(11):e79673. doi: 10.1371/journal.pone.0079673 (PMC3823569; doi:10.1371/journal.pone.0079673)
Supplement: Protocol S1 — Interview on Internet and social network sites (SNS). A detailed description of the variables assessed. (DOC) [file pone.0079673.s001.doc]

**Supporting Information**

Protocol S1 Internet and Social Network Sites (SNS) Interview. A description of variables details. (.DOC)

**Methods:**

**Assessment of Internet use and social network sites:**

The aim of this supporting document is to provide a detailed description of the interviews and approaches used to assess the use of Internet and social network sites (SNS) in our study. Subjects were evaluated using a standard protocol divided into five categories: Social Network, SNS, Facebook, Internet, and Technology. Variables were determined based on the published literature, translated into Portuguese, retro translated into English and adapted by trained researchers in a pilot study. Below we describe each category and the corresponding variables assessed.

1. **Social Network (SNG, SNTW, SNN):**

The Social Network category assessed the number of contacts that an individual had offline (real-world settings) [1, 2, 3]. The Number of Social Network Groups (**SNG**) estimates to how many different social groups (work, family, school) a person is connected; the Number of Social Network in Two Weeks (**SNTW**) estimates the number of offline acquaintances that a person contacts over a two weeks’ period; finally, the Social Network Number (**SNN**) estimates the subject’s lifetime total number of offline contacts. At this stage of the interview, subjects were encouraged to think logically and quantify their contacts. We inquired specifically about the subject’s groups, contacts, and networks.

1. **Social Network Sites (SNS)**

We measured knowledge of SNS by listing terms commonly associated with SNS and assessing the subjects’ familiarity with those terms. At first, patients were asked to inform how many terms of the list they had already heard or used (**SNS terms**) [11]. Terms were retrieved from four SNS: Facebook, LinkedIn, Twitter, and Orkut (the latter was very popular in Brazil from 2004 to 2009). The list comprised the following terms: "update the status," "like," "poke," and "news feed" from Facebook; "recommend" from LinkedIn; "follow," "retweet," and "send DM" from Twitter; and "fake," "testimony," "scrap," and "community" from Orkut. In a second stage, the definitions of each term were read, and subjects were asked to cross-check them with the list of terms. The number of correct answers on this test was used as a score (**Correct SNS**).

The subjects’ age generation, determined by date of birth, was also cross-checked with the characteristics expected for each generation (Millennials or Y, X, and Baby Boomer) according to the literature [4, 5, 6, 9]. According to the data reviewed, Millennials, also called Gen Y (8 terms), know more SNS terms than Gen X (5 terms) and Baby Boomers (1 term). Based on these findings, we determined an expected “Correct SNS” score for each generation. The variable **G-ESNS** was then created to measure the expected pattern of knowledge of SNS according to each subject’s age generation.

Patients and controls were also asked about the number of SNS used (**Number of SNS**) and about the frequency of use of SNS (**Frequency of use of SNS**). They were asked to inform whether or not they used SNS daily and to report number of hours of use. These variables were adapted from the Pew Internet and American Life Project [4, 8, 9].

1. **Facebook**

The main measure in this category was Facebook number of friends (**FBN**), i.e., the number of contacts added to Facebook. This variable was chosen based on perspectival studies that have suggested that these data could reveal information about the use of SNS by the subjects, their online social skills, and even aspects related to the size of brain structures [1, 2, 10]. Participants were also asked to report on their experience using Facebook (**Experience on Facebook**) by answering a questionnaire with seven questions covering their activities in this social medium. Positive responses were scored. Then, they were asked to inform the number of contacts added to Facebook, distinguishing between close contacts (**Close contacts on Facebook**), acquaintances or people seen only once in an offline setting (**Acquaintances on Facebook**), and contacts started online or people never seen in person (**Virtual friendships**). Again at this stage, subjects were encouraged to think logically and quantify their contacts, this time on Facebook. Finally, they were inquired about the frequency of activities on Facebook (**Frequency of Facebook use**). This questionnaire had four questions, with six possible responses each, covering the frequency of activities on Facebook. The final score was determined by summing all answers.

1. **Internet**

In line with the Pew Internet and American Life Project, we also inquired patients and controls about their knowledge and use of the Internet. Their experience with the use of the Internet (**Internet experience**) was assessed using a questionnaire with 13 questions and four possible responses each, all covering the frequency of different activities. The final score was determined by summing all answers. This assessment provided us with a detailed understanding of the activities that the subject knew and about his/her frequency on the Internet. Moreover, participants were asked about their use of the Internet (**Internet use**), i.e., whether they used it or not. The frequency of use (daily or not) was also inquired (**Frequency of Internet use – daily**), as was the number of hours of use.

In line with the approach adopted for **G-ESNS**, the expected frequency of Internet use according to each subject’s generation (**G-EFI**) was measured using scores previously determined based on literature findings. Millennials were expected to use the Internet daily, Gen X at least three times a week, and Baby Boomers one time in two weeks on average.

1. **Technology**

In this category, subjects were asked about their experience with the use of the Internet on digital devices (**Internet experience on devices**). This questionnaire comprised four questions covering devices that they could possibly have used in their lifetime. Positive responses were scored. Finally, we interviewed subjects about the number of digital devices they had ever had (**Digital devices**). This questionnaire comprised eight questions and covered devices that they could possibly have. Positive responses were scored.

***References:***

[1] Cohen S, Doyle WJ, Skoner DP, Rabin BS, Gwaltney JM Jr. (1997) Social ties and susceptibility to the common cold. JAMA 277(24):1940-4.

[2] Bickart KC, Wright CI, Dautoff RJ, Dickerson BC, Barrett LF. (2011) Amygdala volume and social network size in humans. Nature neuroscience, 14 (2), 163-4.

[3] Kanai R, Bahrami B, Roylance R, Rees G. (2011) Online social network size is reflected in human brain structure. Proceedings of the Royal Society B: Biological Sciences, 1-8.

[4] Hampton KN, Goulet LS, Rainie L, Purcell K. (2011) Social Network sites and our lives: How people’s trust, personal relationships, and civic and political involvement are connected to their use of social networking sites and other technologies. PEW Internet & American Life Project/ Pew Research Center. Available: http://pewInternet.org/Reports/2011/Technology-and-social-networks.aspx. Accessed 3rd march, 2013.

[5] Boyd, DM & Ellison, N B (2007). Social Network Sites: Definition, History and Scholarship. Journal of Computer-Mediated Communication, 13 (1), article 11.

[6] Ellison NB, Steinfield C & Lampe C. (2007) The benefits of Facebook “friends”: Social capital and college students’ use of online social network sites. Journal of Computer-Mediated Communication, 12 (4), article 1.

[7] Pew Research Center's Internet & American Life Project. (2011) September 2010 Health Tracking Survey. Accessed in August, 2011. Available:

http://pewInternet.org/Shared-Content/Data-Sets/2010/September-2010--Health.aspx

Accessed 3rd march, 2013.

[8] Pew Research Center's Internet & American Life Project. (2011) September Tracking Survey 2009. Available: http://www.pewInternet.org/~/media/Files/

Questionnaire/2010/PIAL%20September%202009%20and%20TeenParent%20toplines.pdf. Accessed 3rd march, 2013.

[9] Strauss W, Howe, N. (1992) Generations: The History Of America's Future, 1584 to 2069. New York: William Morrow. 545 p.

[10] Stileman E., Bates T. (2007) Construction of the Social Network Score (SNS) Questionnaire for undergraduate students, and an examination of the pre-requisites for large social networks in humans? Unpublished undergraduate thesis.

[11] Tun PA, Lachman ME. (2010) The Association Between Computer Use and Cognition Across Adulthood: Use It So You Won’t Lose It? Psychology and Aging, 25:3, 560 –568.

**Table of Assessment of Internet use and social network sites References**

| **Social network** | SNG | 1,2,3 |
| --- | --- | --- |
|  | SNTW | 1,2,3 |
|  | SNN | 1,2,3 |
| **Social network sites (SNS)** | G-ESNS | 4, 5, 6, 9 |
|  | SNS terms | 4, 11 |
|  | Correct SNS | 4, 11 |
|  | Number of SNS | 4 |
|  | Frequency of use of SNS (daily) | 4 |
| **Facebook** | FBN | 2,3,4,10 |
|  | Experience on Facebook | 8 |
|  | Close contacts on Facebook | 4 |
|  | Acquaintances on Facebook | 4 |
|  | Virtual friendships | 4 |
|  | Frequency of use of Facebook | 4 |
| **Internet** | Internet use (yes) | 4 |
|  | Frequency of Internet use (daily) | 4 |
|  | Experience on Internet | 4 |
|  | G-EFI | 4, 5, 6, 9 |
| **Technology** | Internet experience on devices | 7 |
|  | Digital devices | 8 |

*SNG: lifetime number of social network groups; SNTW: number of social network contacts contacted at least once over two weeks; SNN: social network number or lifetime total number of friends; G-ESNS: expected knowledge of SNS terms according to generation; SNS terms: familiarity with social network site terms; Correct SNS: number of correct answers in the social network site term test; Number of SNS: lifetime number of social network sites used; Frequency of use of SNS: frequency of use of social network sites; FBN: Facebook number of friends; Close contacts on Facebook: close contacts added to Facebook; Acquaintances on Facebook: people seen once added to Facebook; Virtual friendships: people never seen offline but added to Facebook; Frequency of use of Facebook: frequency of activities on Facebook;Frequency of Internet use: frequency of use of the Internet; G-EFI: expected frequency of Internet use according to generation; Digital devices: number of technological devices.*
